# Bioactivities and action mechanisms of active compounds from *Hottuynia cordata* T**hunb** on human lung cancer cells

**DOI:** 10.37796/2211-8039.1219

**Published:** 2021-06-01

**Authors:** Yuh-Fung Chen, Wen-Hsin Chang, Huei-Yann Tsai, Min-Min Lee, Tian-Shang Wu

**Affiliations:** a Department of Pharmacology, China Medical University, Taichung, Taiwan; b Department of Pharmacy, China Medical University Hospital, Taichung, Taiwan; c Department of Food Nutrition and Health Biotechnology, Taichung, Taiwan; d Department of Pharmacy, Cheng Kung University, Tainan, Taiwan

**Keywords:** *Hottuynia cordata* Thunb, Anti-lung cancer, Aristolactam BII, Aristolactam AII, Noraristolodione, Cell cycle arrest, Apoptosis

## Abstract

**Background:**

Lung cancer is the leading cause of death in Taiwan for years. Besides the currently used chemotherapy, herbal medicine may play a role in the treatment of lung cancer. *Hottuynia cordata* Thunb (HC), one of the frequently used herbal medicine in Taiwan, has been widely used in various diseases. Review from literatures, HC has many effects, including anti-inflammatory, anti-viral, anti-bacterial, anti-SARS, and anti-tumor activities. However, there is no literatures describe its active compounds on lung cancer. This present study aims to evaluate the possible effect and action mechanism of active compounds from HC (aristolactam BII, aristolactam AII, and noraristolodione) on lung cancer. A549 lung cancer cell line was used to evaluate the effects of HC on the cell viability and possible anti-tumor effects.

**Methods:**

We used A549 cells in the evaluation of anticancer activity. Cell viability, cell cycle, cell apoptosis and apoptosis related protein expression were studied.

**Results:**

Active compounds from HC significantly inhibited A549 cell viability and induced accumulation of cell cycle at S or G2/M phase on A549 cells in a concentration-dependent manner, and induced A549 arrest at S or G2/M phase via increasing p21, p27, p53 and reducing cyclin-E, -A, cyclin-dependent kinase 2 (CDK2), cdc-2 (CDK1) protein expression. Additionally, HC induced A549 cell late apoptosis by up-regulating caspase-3, -8, Bax and decreasing Bcl-2 protein expression.

**Conclusions:**

The anti-tumor effects of aristolactam BII, aristolactam AII, and noraristolodione on human lung carcinoma A549 cells were via cell cycle arrest and apoptosis.

## 1. Introduction

Lung cancer is the leading cause of cancer death in Taiwan and other countries for years [1]. In many countries in Europe and America, lung cancer is also cancer with the highest mortality rate [2]. How to prevent and effectively treat lung cancer to reduce its incidence and mortality is an important issue.

In addition to current chemotherapy, the use of Chinese herbal medicine may have a reference value for lung cancer [3]. *Houttuynia cordata* Thunb (*Saururaceae;* in abbreviation HC) is a very commonly used herbal medicine in Taiwan. The crude water layer of HC has anti-inflammatory activity [4,5]. It has anti-inflammatory activity against nitric oxide (NO) and tumor necrosis factor-alpha (TNF-α) in the RAW264.7 cell line [3]. HC injection can inhibit carrageenan-induced pleurisy in rats and xylene-induced ear edema in mice [5]. The polyphenols in HC have antioxidant effects. For example, flavonoids can decompose the free radicals generated by oxidative stress in patients with diabetes [4–6]. Water extract of HC protects against bleomycin-induced pulmonary fibrosis [7]. HC can reduce viral plaque formation and reduce viral RNA synthesis, increase viral apoptosis [8]; it can also reduce the replication of herpes virus (HSV-2) achieve anti-viral infection activity [9]. Hot water extract of HC can effectively inhibit the growth of human leukemia cells [10].

Water layer extract of HC can stimulate the proliferation of mouse spleen lymphocytes by increasing the secretion of IL-2 and IL-10 of visceral lymphocytes, and at the same time increase the CD4^+^ and CD8^+^ T cells of the immune system; in terms of anti-virus, it shows inhibitory effects on SARS-CoV 3C-like protease (3CLpro) and RNA-dependent RNA polymerase (RdRp) [11]. Six active alkaloids derived from the methanol extraction of HC are aristolactam BII, piperolactam A, aristolactam AII, norcepharadione B, cepharadione B, and splendidine, which can effectively inhibit the growth of five human cancer cell lines. Non-small cell lung cancer (A549), ovarian cancer (SK-OV-3), melanoma (SK-MEL-2), central nervous system cancer cells (XF-498) and colon cancer (HCT-15) are included [12,13]. However, there are no reposts regarding the action mechanisms of these active alkaloids on human lung cancer.

This study evaluated the possible effect and action mechanism of active alkaloids from HC on lung cancer. We used the A549 lung cancer cell line to evaluate HC’s effects on cell viability and possible anti-tumor effects.

## 2. Materials and Methods

### 2.1. Active alkaloids from HC

Five active alkaloids derived from the methanol extraction of *Houttuynia cordata* (HC), aristolactam BII, aristolactam AII, piperolactam A, norcepharadione B, and noraristolodione were prepared and identified by Prof. Wu TS’s lab.

### 2.2. Chemicals and reagents

RPMI cell culture medium, FBS (fetal bovine serum), P/S (penicillin-streptomycin), and trypan blue were purchased from Gibco BRL USA. DMSO (dimethyl sulfoxide), PI (propidium iodide), RNase A (ribonuclease A), BSA (bovine serum albumin), APS (ammonium persulfate), MTT (3-(4,5-dimethylthiazol-2-yl)-2,5-diphenyl-tetrazolium bromide), Cellytic ™ M cell lysis reagent, Triton X-100 were from Sigma-Aldrich Chemical company, USA. TEMED (N–N–N′-N′-Tetramethylenediamine), Tween-20 (polyoxyethylenesorbitan monolaurate), trypran-EDTA, 40% acrylamide/BIS-ACRYLAMIDE (29:1 Ratio), 10X SDS (sodium dodecylsulfate) buffer, Tris (tris(hydroxymethy)-aminomethane), 10X SDS-PAGE running buffer (TG-SDS buffer) were from Amersco company, USA. Apoptosis detection kit (PI/Annexin V-FITC) was from Becton Dickinson, USA. Western lightning chemiluminescene reagent plus (ECL) kit was from NEN Life Science, USA. Anti-cyclin A (ab7956), anticyclin E (ab7959), anti-CDk1/Cdc2 (ab6537), anti-Cdk2 (ab7954), Anti-p27K1P1 (ab47590), Anti-p21 (ab47452), goat anti-mouse IgG (HRP) horseradish peroxide conjugated antibody were from Abcam company. β-Actin (C4): sc-477778, caspase-3 (H-277): sc-7418, caspase-8 p20 (H-134):sc-7890, were from Santa Cruz biotechnology.

### 2.3. Cell culture

Human lung cancer A549 cells were from BCRC (the Bioresource Collection and Research Center) in Hsinchu, Taiwan. A549 cells were maintained in RPMI-1640 medium containing 100 ml/L FBS and 100,000 U/L penicillin/100 mg/L streptomycin.

### 2.4. Cell viability

A549 cells were plated onto 96-well plates and incubated with different concentrations (12,5 μg/ml, 25 μg/ml, 50 μg/ml) of five alkaloids from HC (including aristolactam BII, aristolactam AII, piperolactam A, norcepharadione B, and noraristolodione) for 24 hr. MTT was added to each well and incubated for another 1 h at 37°C. Dissolved the blue formazan product in 200 μl DMSO for 15 min, and then the plates were read using a spectrophotometric plate reader (Bio-Rad, Japan) at O.D. 570nm.

### 2.5. Cell cycle distribution and apoptosis determination

A549 cells were plated onto 12-well plates and incubated with different concentrations (12,5 μg/ml, 25 μg/ml, 50 μg/ml) of 3 alkaloids from HC (including aristolactam BII, aristolactam AII, and noraristolodione) for 24 hr. Fixed cells in 75% ethanol at 4 °C for overnight and resuspended in 1X PBS containing PI (40 μg/ml), RNase (0.1 mg/ml) and Triton X-100 (0.1%) for 30 min. Cell cycle distribution and apoptosis determination were analyzed by flow cytometry (FACS Calibur, Becton Dickinson, USA) as previously described [13].

### 2.6. Western blotting

A549 cells were plated onto 10-cm plates and treated with various concentrations (12,5 μg/ml, 25 μg/ml, 50 μg/ml) of aristolactam BII, aristolactam AII, and noraristolodione for 24 hr. Total cell lysates were prepared as previously described [13]. Applied forty μg of total protein to SDS-PAGE and transferred onto a PVDF (polyvinylidene fluoride) membrane. Then, incubated the blots with the appropriate dilution of specific monoclonal antibodies for cyclin E, cyclin A, CDK2, cdc 2, p21, p27, p53, caspase 3, caspase 8, Bax and Bcl-2. Using enhanced chemiluminescence kits (ECL kit) to detect protein expressions [13].

### 2.7. Statistical analysis

The experiments were performed at least in triplicate and all data were expressed as the mean ± standard error. Student’s *t*-test and one-way ANOVA followed by Dunnett’s test were used for single variable comparison and multiple variable comparisons, respectively. Significance was considered when *P* < 0.05.

## 3. Results

### 3.1. Effects of alkaloids from HC on the viability of A549 cells

**Fig. 1:**
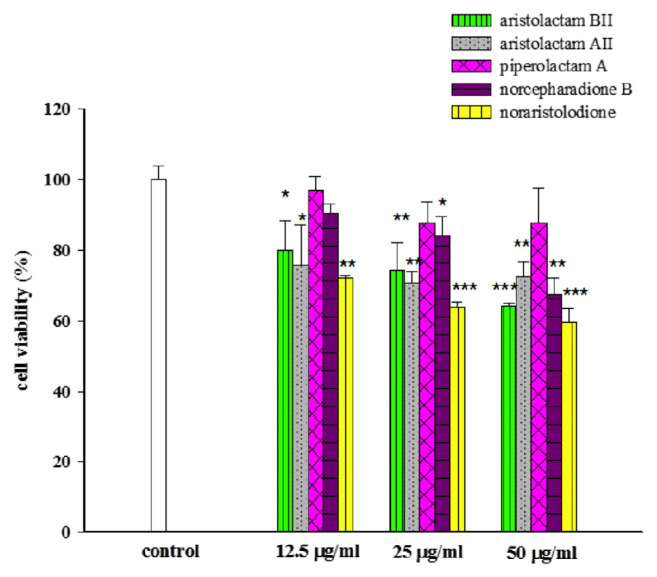
Effects of active compounds (aristolactam BII, aristolactam AII, piperolactamA, norcepharadione B, and noraristolodione) on cell viability of human lung cancer A549 cells. Various concentrations (12.5 μg/ml, 25 μg/ml, 50 μg/ml) of these active compounds treated with A549 cells for 24 h. Detected the cell viability by MTT tests. Results were represented as vertical bars and mean ± S.E. *P < 0.05, **P < 0.01, ***P < 0.001 was considered statistically significant compared to the control group.

Various doses (12.5 μg/ml, 25 μg/ml, 50 μg/ml) of active compounds (aristolactam BII, aristolactam AII, piperolactam A, norcepharadione B, and noraristolodione) from HC treated with A549 cells for 24 h, and the cell viability was detected by MTT tests. These compounds showed a dose-dependent inhibition on the cell viability of human lung cancer A549 cells, *P < 0.05, **P < 0.01, and ***P < 0.001 (as shown in Fig. 1).

### 3.2. Effects of alkaloids from HC on the cell cycle of A549 cells

**Fig. 2:**
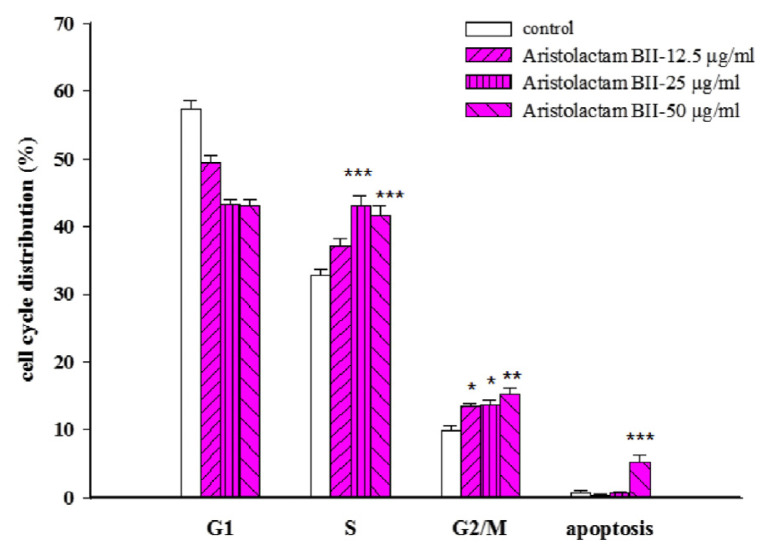
Effects of different concentrations (12.5 μg/ml, 25 μg/ml, and 50 μg/ml) of aristolactam BII on A549 cell cycle distribution. Aristolactam BII treatment for 24 h altered A549 cell-cycle distribution and arrested at S/G2/M phase. Results were represented as vertical bars and mean ± S.E. *P < 0.05, **P < 0.01, ***P < 0.001 was considered statistically significant compared to the control group.

**Fig. 3:**
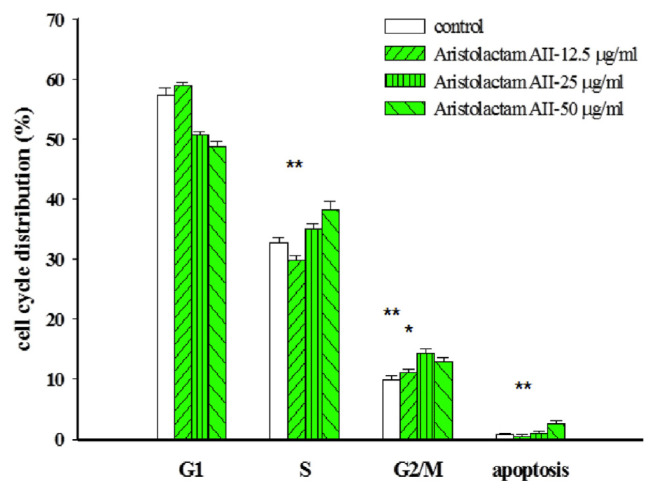
Effects of different concentrations (12.5 μg/ml, 25 μg/ml, and 50 μg/ml) of aristolactam AII on A549 cell cycle distribution. Aristolactam AII treatment for 24 h altered A549 cell-cycle distribution and arrested at S/G2/M phase. Results were represented as vertical bars and mean ± S.E. *P < 0.05, **P < 0.01, ***P < 0.001 was considered statistically significant compared to the control group.

**Fig. 4:**
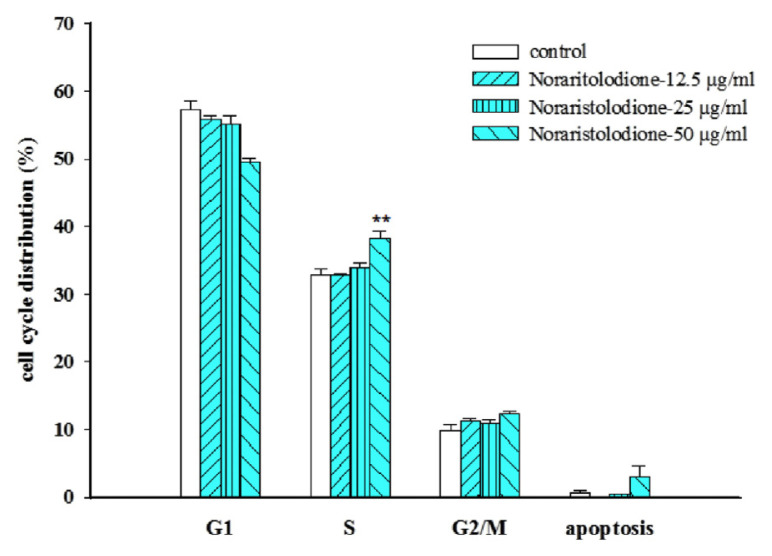
Effects of different concentrations (12.5 μg/ml, 25 μg/ml, and 50 μg/ml) of noraristolodione on A549 cell cycle distribution. Noraristolodione treatment for 24 h altered A549 cell-cycle distribution and arrested at S/G2/M phase. Results were represented as vertical bars and mean ± S.E. *P < 0.05, **P < 0.01, ***P < 0.001 was considered statistically significant compared to the control group.

The DNA content of the cell cycle is highest in the G1 phase, followed by the S phase, and the G2/M phase is the least. Compared to the control group, the alkaloids aristolactam BII and aristolactam AII produced the S phase and G2/M phase accumulation (*P < 0.05, **P < 0.01, and ***P < 0.001, as shown in Fig. 2 and Fig. 3), and noraristolodione only caused accumulation in the S phase (**P < 0.01, as shown in Fig. 4)

### 3.3. Effect of alkaloids from HC on the cell cycle-related and apoptosis-related proteins of A549 cells

**Fig. 5:**
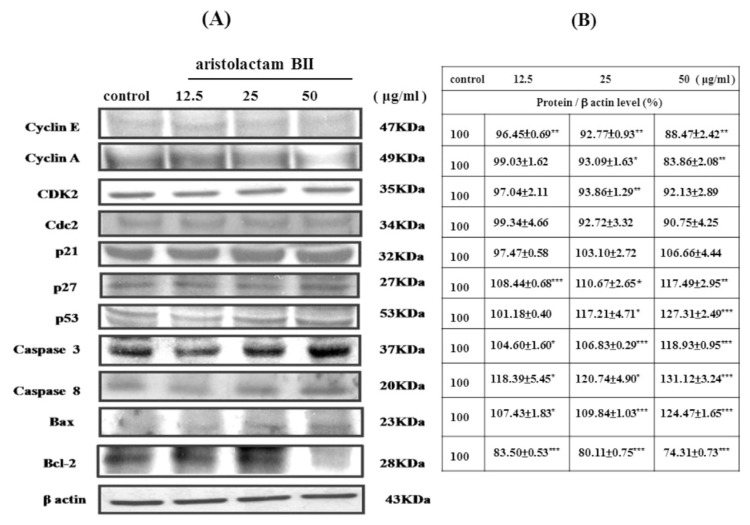
Various concentrations (12.5 μg/ml, 25 μg/ml, and 50 μg/ml) of aristolactam BII treated A549 cells for 24 h suppressed CDKs, cyclins, Bcl-2 protein expression and up-regulation of apoptotic protein (caspase 3, caspase 8, p21, p27, p53, and Bax) expression (A). Protein/β actin level (%) presented in (B). The experiments were done in triplicate with similar results and used the blot of β actin as a loading control. *P < 0.05, **P < 0.01, ***P < 0.001 was considered statistically significant compared to the control group.

**Fig. 6:**
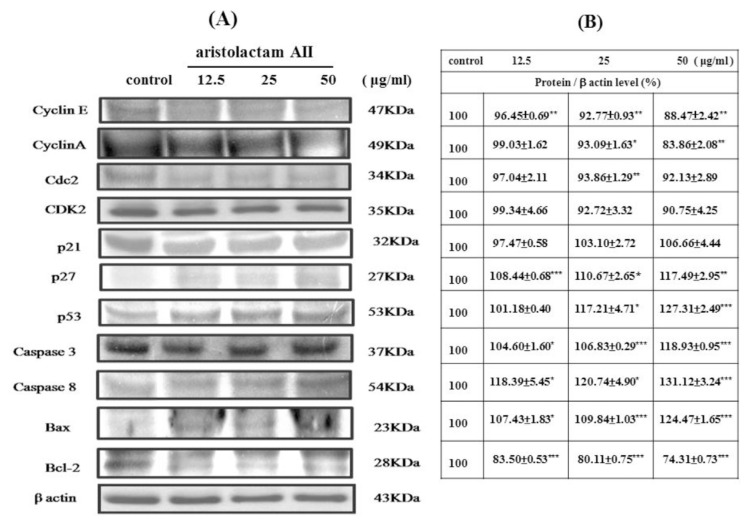
Various concentrations (12.5 μg/ml, 25 μg/ml, and 50 μg/ml) of aristolactam AII treated A549 cells for 24 h suppressed CDKs, cyclins, Bcl-2 protein expression and up-regulation of apoptotic protein (caspase 3, caspase 8, p21, p27, p53, and Bax) expression (A). Protein/β actin level (%) presented in (B). The experiments were done in triplicate with similar results and used the blot of β actin as a loading control. *P < 0.05, **P < 0.01, ***P < 0.001 was considered statistically significant compared to the control group.

**Fig. 7:**
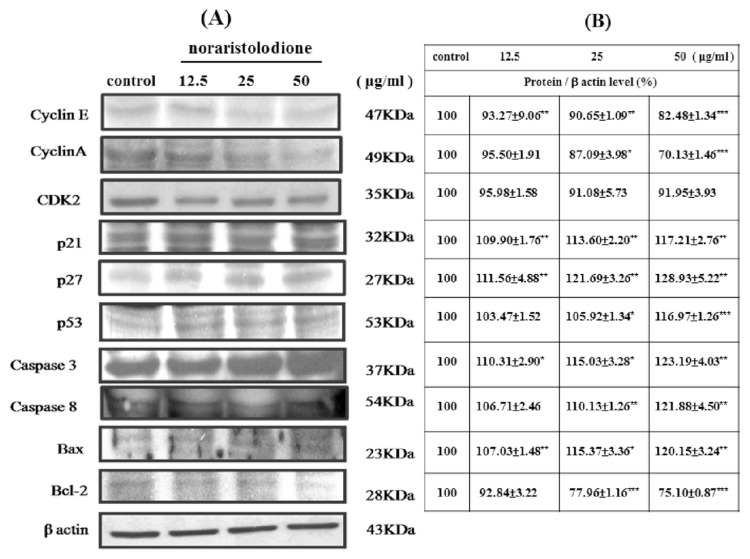
Various concentrations (12.5 μg/ml, 25 μg/ml, and 50 μg/ml) of noraristolodione treated A549 cells for 24 h suppressed CDKs, cyclins, Bcl-2 protein expression and up-regulation of apoptotic protein (caspase 3, caspase 8, p21, p27, p53, and Bax) expression (A). Protein/β actin level (%) presented in (B). The experiments were done in triplicate with similar results and used the blot of β actin as a loading control. *P < 0.05, **P < 0.01, ***P < 0.001 was considered statistically significant compared to the control group.

Alkaloids (aristolactam BII, aristolactam AII, and noraritoldione) from HC decreased the cyclin A, cyclin E, CDK 2, and Cdc2 (CDK1) proteins related to the S phase and G2/M phase of the cell cycle in a dose-dependent manner (*P < 0.05, **P < 0.01, and ***P < 0.001, as shown in Figs. 5–7). Alkaloids from HC concentration-dependent increased the apoptosis-related protein p21, p27, P53, caspase 3 and caspase 8, and Bax. However, the survival protein Bcl-2 was reduced (*P < 0.05, **P < 0.01, and ***P < 0.001, as shown in Figs. 5–7).

## 4. Discussion

According to previous reports, *Houttuynia cordata (*HC) does affect some human cancer cell lines, such as non-small cell lung cancer (A549 cells), ovarian cancer (SK-OV-3 cells), and melanoma (SK-MEL-2 cells) [12,13]. HC has anti-growth effects on central nervous system cancer cells (XF-498 cells) and colon cancer (HCT-15 cells) [12]. HC has anti-lung cancer activity by interfering cell cycle; it modulates G0/G1 arrest and stimulates the Fas/CD95 protein level [13]. Thus, leads to caspase-8 and caspase-3 activation and resulting in the induction of apoptosis in human lung cancer A549 cells [13]. At present, chemotherapy and radiotherapy are mostly used for the treatment of lung cancer in clinical practice [14,15], all of which have side effects. In order to provide a reference for the clinical application of HC, the anti-cancer mechanism of active compounds from HC is studied.

Previous results show HC extract modulates G_0_/G_1_ arrest and Fas/CD95-mediated death receptor apoptotic cell death in human lung cancer A549 cells [13]. The mechanism of HC extract on A549 cells is reducing the expression of cyclin D1, cyclin E, cyclin A, and CDK4/CDK6 and CDK2 proteins in the cell cycle, and increasing the expression of p27KIP1 Arrest A549 cells in the G1/S phase, and activate the external pathways of caspase 3 and caspase 8 to promote cell apoptosis [13].

There were about 44 compounds isolated from the whole plant of HC [16]. Based on the toxic effect of the crude extracts on A549 cells, it may be derived from the alkaloids contained in HC. After testing five kinds of alkaloids from HC revealed that three of them have a significant inhibitory effect on A549 cells. Treated A549 cells with different concentrations of these alkaloids revealed a decrease of the survival rate (as shown in Fig. 1). These three alkaloids, aristolactam BII, aristolactam AII and noraristolodione, were used to explore their effects on A549 cells. Under the treatment of aristolactam BII and aristolactam AII, A549 cells were found to accumulate in S phase or G2/M phase, that is, cell cycle growth stopped in S phase or G2/M (as shown in Figs. 2 and 3). Under the treatment of noraristolodione, the cell cycle of A549 cells was arrested in S phase (as shown in Fig. 4), and treated these alkaloids at 50 μg/ml will promote A549 cell apoptosis.

The protein expression level results revealed that aristolactam BII and aristolactam AII dose-dependently decreased the cyclin E, cyclin A, CDK 2, and Cdc 2 proteins in the S phase or G2/M phase. However, aristolactam BII and aristolactam AII dose-dependently increased the p21, p27, and p53 of the cell cycle protein, and the apoptosis-related proteins caspase 3, caspase 8, Bax. Aristolactam BII and aristolactam AII dose-dependently decreased Bcl-2 expression level, as shown in Figs. 5 and 6. As to noraristolodione, it dose-dependently inhibited cyclin E, cyclin A, CDK 2, and dose-dependently decreased the p21, p27, p53 of the cell cycle protein. It dose-dependently increased caspase 3, caspase 8, Bax protein level and dose-dependently decreased Bcl-2 expression (as shown in Fig. 7). p53 is a transcription factor that is responsible for regulating the balance between normal cell growth and apoptosis. There are a large number of variability p53 in cancer cells [17,18]. According to the experimental results, the alkaloids in HC can inhibit the activity of A549 cells by influencing cell cycle-related proteins and apoptosis-related proteins. It may be that p21/p27 increase by activating p53 on the one hand, allowing A549 cell cycle arrest; on the other hand, by increasing the expression of Bax, caspase 8, and caspase 3 and reducing Bcl-2 activity [19].

## 5. Conclusions

**Fig. 8:**
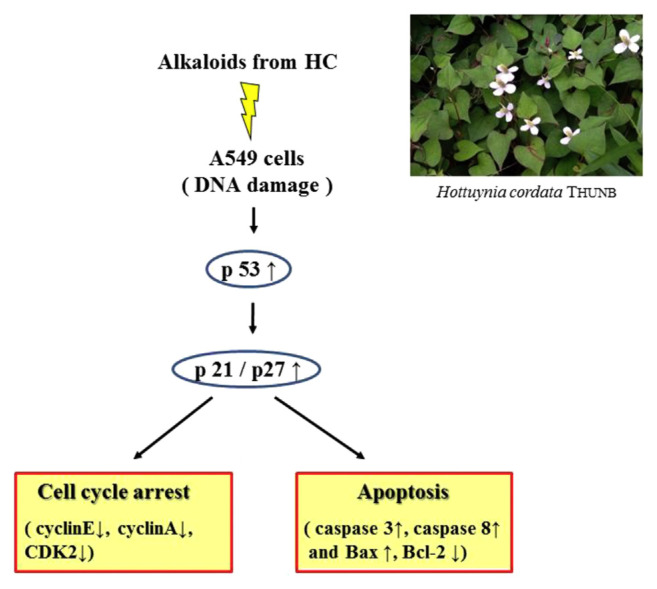
Proposed model of alkaloids from Hottuynia cordata Thunb (in abbreviation HC) modulates S/G2/M arrest and induces apoptotic cell death on human lung cancer A549 cells.

From the above results, alkaloids from HC reduced cyclin E, cyclin A, CDK2, and Cdc2 (CDK1) of the cell cycle of A549 cells, and increased the expression of p21cip, p27KIP1, and p53 proteins, so that A549 cells were stagnant. In the S or G2/M phase, while increasing caspase 3, caspase 8, Bax and reducing the protein expression of Bcl-2. A549 cells underwent apoptosis through internal and external pathways. The anti-tumor effects of aristolactam BII, aristolactam AII, and noraristolodione on human lung cancer A549 cells were via cell cycle arrest and apoptosis (as shown in Fig. 8).
